# Temperature mediates continental-scale diversity of microbes in forest soils

**DOI:** 10.1038/ncomms12083

**Published:** 2016-07-05

**Authors:** Jizhong Zhou, Ye Deng, Lina Shen, Chongqing Wen, Qingyun Yan, Daliang Ning, Yujia Qin, Kai Xue, Liyou Wu, Zhili He, James W. Voordeckers, Joy D. Van Nostrand, Vanessa Buzzard, Sean T. Michaletz, Brian J. Enquist, Michael D. Weiser, Michael Kaspari, Robert Waide, Yunfeng Yang, James H. Brown

**Affiliations:** 1 State Key Joint Laboratory of Environment Simulation and Pollution Control, School of Environment, Tsinghua University, Beijing 100084, China; 2 Institute for Environmental Genomics, Department of Microbiology and Plant Biology and School of Civil Engineering and Environmental Sciences, University of Oklahoma, Norman, Oklahoma 73019, USA; 3 Earth Science Division, Lawrence Berkeley National Laboratory, Berkeley, California 94270, USA; 4 CAS Key Laboratory for Environmental Biotechnology, Research Center for Eco-Environmental Science, Chinese Academy of Sciences, Beijing, 100085, China; 5 Department of Ecology and Evolutionary Biology, University of Arizona, Tucson, Arizona 85721, USA; 6 The Santa Fe Institute, USA, 1399 Hyde Park Rd, Santa Fe, New Mexico 87501, USA; 7 EEB Graduate Program, Department of Biology, University of Oklahoma, Norman, OK 73019, USA; 8 Smithsonian Tropical Research Institute, Balboa 0843-03092, Republic of Panama; 9 Department of Biology, University of New Mexico, Albuquerque, New Mexico 87131, USA

## Abstract

Climate warming is increasingly leading to marked changes in plant and animal biodiversity, but it remains unclear how temperatures affect microbial biodiversity, particularly in terrestrial soils. Here we show that, in accordance with metabolic theory of ecology, taxonomic and phylogenetic diversity of soil bacteria, fungi and nitrogen fixers are all better predicted by variation in environmental temperature than pH. However, the rates of diversity turnover across the global temperature gradients are substantially lower than those recorded for trees and animals, suggesting that the diversity of plant, animal and soil microbial communities show differential responses to climate change. To the best of our knowledge, this is the first study demonstrating that the diversity of different microbial groups has significantly lower rates of turnover across temperature gradients than other major taxa, which has important implications for assessing the effects of human-caused changes in climate, land use and other factors.

## Abstract (web summary)

Climate warming has a wide range of effects on biodiversity. Here, Zhou *et al*. show that although variation in environmental temperature is a primary driver of soil microbial biodiversity, microbes show much lower rates of turnover across temperature gradients than other major taxa.

## Graphical abstract

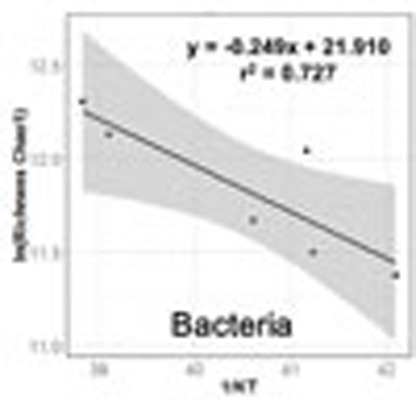

Climate change is the largest anthropogenic disturbance on natural systems^1^,^2^, and predicting the response of the biosphere to shifting climate is one of the most formidable scientific and political challenges in the 21st century^3^,^4^. Since 1850, the Earth's surface temperature has increased by 0.76 °C and is expected to increase by another 1.1– 6.4 °C by the end of this century^2^. As a result of climate warming, precipitation patterns at regional and global scales are and will likely continue to be altered. This magnitude of climatic change and its induced extreme climatic events will profoundly affect ecosystem functions and most likely reduce ecosystem services. Climate change has profound and diverse effects on all levels of biological organization from individual organisms to whole-biome levels^5^. However, the lack of a general theoretical framework to mechanistically and quantitatively link how variation in abiotic environment will influence variation in ecological processes across all taxa limits our ability to predict future impacts within and across diverse taxa^6^.

Since temperature directly accelerates metabolic rates and biochemical processes^7^,^8^, a promising framework for linking abiotic environmental changes to communities is to assess how temperature affects organismal metabolism and then influences their ecology and evolution. The metabolic theory of ecology (MTE)^7^,^8^ starts from first principles of biophysics to scale up the kinetic effects of temperature on metabolism to rates of evolution, community structure, gradients of diversity and ecosystem processes^9^. In general, MTE predicts that the metabolism of individuals, the population growth rate and the number of species increase exponentially with the environmental temperature (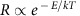)^7^,^10^, where *R* is the rate of some process such as metabolism, population growth or speciation, *e* is the base of the natural logarithm, *E* is the ‘activation energy' that characterizes the temperature dependence of a given biological process, *k* is Boltzmann's constant and *T* is temperature in kelvin. Rates of ecological and evolutionary processes, including rates of population growth, species interactions, mutation and speciation, are all predicted to have approximately the same exponential temperature dependence as metabolic rate, with an ‘activation energy' of ∼0.65 eV, equivalent to a *Q*_10_ of ∼2.5 (ref. 10).

Because the changes in ecosystem process rates are ultimately dependent on the changes in metabolic demands of individual organisms, it is expected that temperature should have a profound influence on ecological community structure, and hence MTE should provide a powerful framework for predicting the effects of climate warming on biodiversity and ecosystem processes^6^,^7^,^11^. However, despite a decade of intensive research, empirical^11^,^12^ and theoretical evaluations^13^ of MTE have produced variable, but seemingly contradictory results^9^,^14^. Further, most assessments of MTE predictions have focused on plants and animals^7^. As a result, a recent paper has questioned its theoretical foundations and empirical validity^9^. Clearly, to better enable predictive power of the consequences of forthcoming climate change across all domains of life, there is a strong need for a more complete understanding of the relationships between temperature, diversity and ecological processes in both macrobes and microbes^9^.

Elucidating the drivers of how biological diversity varies over space, time and environmental gradients remain as a central focus in biodiversity science. It is well documented that the microbial diversity under natural settings, particularly in soils, is extremely high, but the mechanisms controlling such high diversity are poorly understood^15^,^16^. Various studies of soil microbes suggest that pH, rather than temperature, plays an important role in shaping microbial community structure^17^,^18^,^19^. A recent study demonstrated that the temperature drives continental-scale distribution of two key cyanobacteria in topsoil^20^, implying that the variation in temperature could underlie community-level microbial diversity and distribution. However, the importance of temperature in shaping microbial community structure in general and the applicability of MTE to soil microbial communities remain unknown. Therefore, in comparison with the known temperature gradients in diversity across macrobes, we ask: (i) does temperature play a major role in shaping continental-scale soil microbial community diversity? and (ii) if so, can MTE be used to predict the diversity patterns of the microbial communities in the forest soils? To address these questions, we used next-generation sequencing technology to analyse 126 soil samples from forest sites of different latitudes in North America, with a wide range of temperature gradient from alpine coniferous forest in Colorado to evergreen tropical forest in Panama. Our results indicate that the temperature is a primary driver in shaping soil microbial community in the forest soils and microbial groups has significantly lower rates of turnover across the temperature gradients than plants.

## Results

### Site characteristics

**Figure 1:**
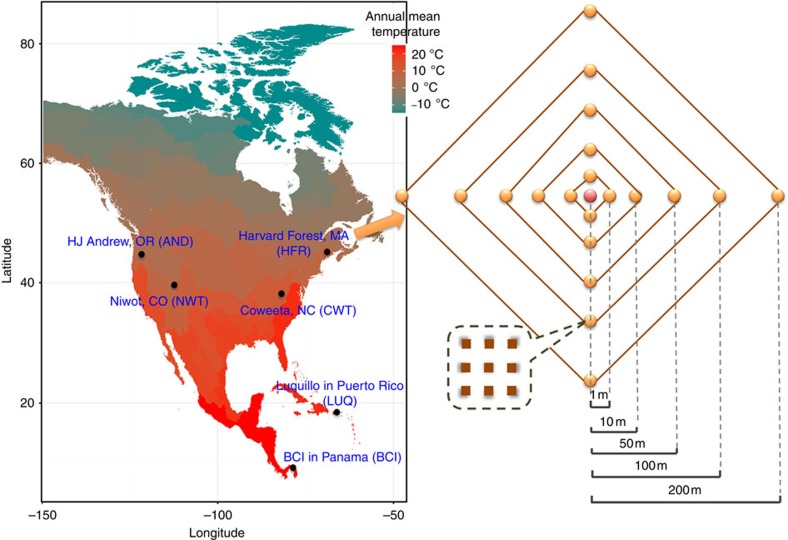
Sampling strategy with nested design. Samples were taken from six forest sites from North America. At each site, 21 nested samples were collected at distance of 1, 10, 50, 100 and 250 m. At each sample point (1 × 1 m), nine soil cores were collected and composited for microbial and soil analysis.

To determine whether temperature is important in controlling microbial community diversity as predicted by MTE, we undertook a continental-scale survey of the microbe diversity in soil samples from six forest sites in North America, with both small (metres)- and large (thousand kilometres)-scale spatial variations (Fig. 1). Since soil environment is highly heterogeneous, the detection of the effects of environmental factors on microbial diversity could be masked. We organized our sampling design to quantify the relative role of small- and large-spatial scales. The distribution of microbial taxa can be locally adapted to edaphic characteristics at the scale of only a few metres^21^. Taken that high dispersal rates of microbes may not trump local processes via mass effects^22^ and that the local niche-based processes may play an important role in determining the patterns of microbial species richness^23^, we sampled microbial communities at multiple spatial scales. Thus, different from several previous studies^17^,^18^,^19^, a total of 21 soil samples from each forest site were collected at metre-scale using a nested design (Fig. 1). Each sample consisted of nine soil cores evenly collected from 1-m^2^ area (Fig. 1). As observed in previous ecosystem diversity–stability studies^24^, such multiple replicate sampling within a site enables us to better quantify the temperature effects on microbial biodiversity across sites and the role of local environmental heterogeneity. Further, nested sampling within and between sites enables us to assess the relative role of small-scale and large-scale processes in shaping the variation in microbial diversity. Consequently, this trade-off in sampling results in fewer among-site comparisons, but many more samples within each site.

These six forested sites spanned the global range of mean annual temperatures experienced by forested ecosystems (from 2.5 to 25.7 °C, and in latitude from 9–44° N (Supplementary Table 1)). Other site characteristics such as annual mean precipitation, soil types, soil moisture, average soil pH, soil total carbon (C) and nitrogen (N) also spanned a large fraction of the global range (Supplementary Table 1). In addition, substantial differences were observed in plant types, from boreal to tropical forest, and plant diversity (Supplementary Table 1). Pairwise comparisons showed that there were strong correlations among latitudes, annual mean temperature and annual precipitation (Supplementary Table 2). Both plant richness and diversity were positively correlated with annual mean temperature and annual precipitation (Supplementary Table 2). However, such covariations present difficulties in discerning the effects of single environmental factors, such as temperature and pH on biological communities (see below).

### Sequencing targets and statistics

To determine the biodiversity of microbial communities, DNA was extracted and purified from each soil sample, and three genes targeting different taxonomic groups and with different taxonomic resolutions were amplified and sequenced using Illumina MiSeq: (i) V4 region of the 16S ribosomal RNA (rRNA) genes for bacteria and archaea; (ii) internal transcribed spacer (ITS) between 5.8S and 28S rRNA genes for fungi; and (iii) bacterial nitrogenase subunit H (*nifH*) for nitrogen-fixing bacteria. Diversity of prokaryotic bacteria and archaea is still being assessed, but increasingly being shown to be enormous^25^. Among eukaryotic organisms, fungi rank second in diversity after arthropods (insects). N fixation is critical in global biogeochemical cycling in providing a major source of nutrients. Since, N-fixing bacteria are only small components of soil microbial communities (comprising perhaps <0.5% of the genomes), surveying *nifH* provides higher resolution than the 16S rRNA genes used to assess the overall bacterial diversity.

An average of 64,000±18,000, 39,000±11,000 and 30,000±9,000 sequence reads per sample were obtained for 16S, ITS and *nifH* genes, respectively (Supplementary Table 3). This level of sequencing appeared sufficient for estimating the diversity of soil microbial communities sampled here, as indicated by rarefaction curves that approached saturation at different cutoffs for the three target genes (Supplementary Fig. 1). Nonetheless, our analysis also indicated that we could have missed the many rare genotypes that occur at all of our study sites (Supplementary Fig. 1). The numbers of OTUs (operational taxonomic units) estimated with these sequences were quite high (Supplementary Table 4). For instance, with resampling, there were ∼190,000 and 17,000 OTUs based on 16S and ITS sequences, respectively, at 97% identity cutoff, and ∼87,000 OTUs based on *nifH* sequences at 95% identity cutoff. Our numbers of OTUs varied considerably (1.3–2.4 times) among these 126 samples based on different sequence similarity thresholds (Supplementary Table 4). These results suggested that the diversity of bacteria, archaea, fungi and N-fixing bacteria was very high in these forest soils.

### Temperature-dependent latitudinal diversity patterns

Although the latitudinal diversity pattern, whereby species diversity tends to decrease as latitude increases, is well documented and intensively studied in plant and animal ecology^26^, it is less clear whether microbes exhibit similar latitudinal diversity gradients^17^,^27^. To determine the latitudinal diversity patterns, the relationships between taxonomic diversity and latitudes were examined. Both taxon richness and Shannon diversity based on these three target genes were strongly correlated with latitude (Supplementary Table 5), indicating that the strong latitudinal diversity patterns exist for bacteria, fungi and N-fixing bacteria.

**Figure 2:**
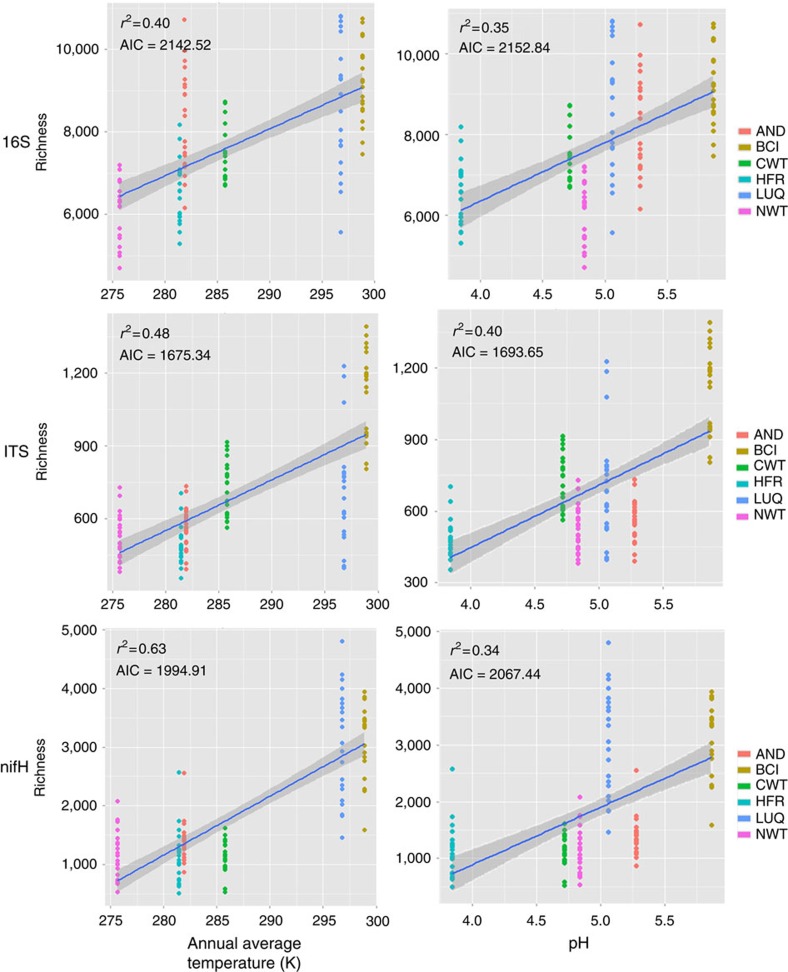
Relationships between taxon richness, and key soil and climate variables. For all plots, the *y* axis is the taxon richness of observed OTUs for 16S, ITS or *nifH* data sets. Three plots in left showed the linear relationships between annual mean temperature (kelvin) and taxon richness of 16S, ITS and *nifH*, respectively, and three plots in right showed the relationships between pH and taxon richness. Line in each plot represents least squares regression fit and the shaded area represents its 95% confidence limits. The relationships of taxon richness with other soil and climate variables were seen in Supplementary Fig. 2. The relationships of microbial biodiversity (taxon richness, Shannon diversity and phylogenetic diversity) to all soil, climate and plants were summarized in Supplementary Table 6.

Further statistical analysis revealed that the microbes in these forest soils exhibited strong correlations with environmental temperature (Fig. 2; *r*^2^=0.40–0.63). The observed continental-scale diversity patterns across the temperature gradient could also reflect the influence of other environmental variables. Of all soil and site variables examined, annual mean temperature, pH, annual precipitation, and tree species richness were all correlated with taxonomic diversity (taxon richness, Shannon diversity and inverse Simpson diversity) and phylogenetic diversity (Faith D and NRI, net relatedness index; Fig. 1; Supplementary Fig. 2; Supplementary Table 6; Supplementary Table 7), indicating that these factors could play important roles in shaping microbial community diversity and structure. In most cases, variation in mean annual temperature explained a greater proportion of variation in microbial diversity, regardless of the regression techniques applied (Fig. 2; Supplementary Table 6; Supplementary Table 7). It should be noted that the effects of temperature on microbial communities could be also exerted indirectly through plants and precipitation because temperature has strong correlations with plants and precipitation (Supplementary Table 2). However, it is not possible to disentangle the direct and indirect effects of temperature on microbial communities because these factors are highly correlated (Supplementary Table 2). One possible way to parse out the indirect effects of plant diversity from direct effects of temperature on microbial diversity is to examine ecosystems along a temperature gradient, in which plant diversity is identical or similar, although it is difficult to identify such ecosystems under natural settings. In addition, assessing covariation between temperature, precipitation, plant diversity and additional variables, including pH, precipitation and their interactions indicates that temperature still plays a primary role in predicting variation in microbial diversity (Supplementary Table 8). The addition of multiple covariates, in addition to temperature only slightly improved the fraction of variation in taxon richness explained, with higher *r*^2^ and lower Akaike information criterion (AIC) (Supplementary Table 8). Together, these results suggest that mean annual temperature plays a major role in shaping variation in the composition and structure of these forest microbial communities.

Previous studies of soil microbes suggest that pH, rather than temperature, plays an important role in shaping community structure^17^,^18^,^19^. To further assess the associations with temperature and pH, we performed partial Mantel tests on the soil variables selected by BIO-ENV^28^: temperature, pH, total C and total N. Our results showed that all three genes had considerably higher correlations with temperature than pH (Table 1). Particularly, for 16S gene, correlations with temperature (*r*_M_=0.674) were more than three times as strong as with pH (*r*_M_=0.179; Table 1), indicating that temperature played a more important role than pH in shaping bacterial community structure. Collectively, all of our results point to temperature, as the strongest correlate of taxon richness and diversity of the forest soil microbial communities (bacteria, fungi, and N fixers) as measured by these three genes.

Variation in temperature could affect the microbial biodiversity through a variety of mechanisms. There are several distinct direct and indirect mechanisms by which increasing temperature can generate and maintain higher species diversity. First, the most important direct mechanism is that higher temperatures lead to higher rates of metabolism, growth rates and population doubling times^7^. These biological rates set the pace of life and underlie near all biological activities at all levels of biological organization^7^,^12^,^27^,^29^. Second, in terrestrial environments, higher temperatures are generally associated with higher rates of ecosystem productivity and hence more species can be supported^7^,^12^. Third, higher temperatures lead to more plant species. Higher plant diversity could provide more substrates, nutrients and/or physical attachments to microorganisms, and thus higher microbial diversity^30^. Our data support such a linkage, as we show strong significant correlations were observed between microbial taxa richness and plant species richness (*r=*0.55–0.81). Fourth, temperature also affects species interactions. Higher temperatures could lead to higher rates of ecological interactions (for example, rates of parasitism, predation and competition)^7^,^31^, which then differentially affects species diversity. In addition, higher temperatures are associated with high rates of evolutionary processes such as mutations and speciation^32^,^33^. For instance, very strong positive correlations have been noted between latitudinal diversity gradients and speciation rates^32^, suggesting the importance of temperature in generating species composition and structure. Finally, temperature could interact with other environmental factors, such as water availability, carbon and nutrient availability, and pH to indirectly affect biodiversity. For instance, warmer environments are often wetter, which is then associated with higher primary production^12^. Higher temperatures cause faster decompositions and hence affect the nutrient availability^4^,^34^, which could support and maintain higher plant and microbial diversity. Discerning these various temperature-dependent mechanisms that can potentially shape microbial diversity is very challenging, particularly under natural soil environmental conditions.

### Metabolic theory of ecology

**Figure 3:**
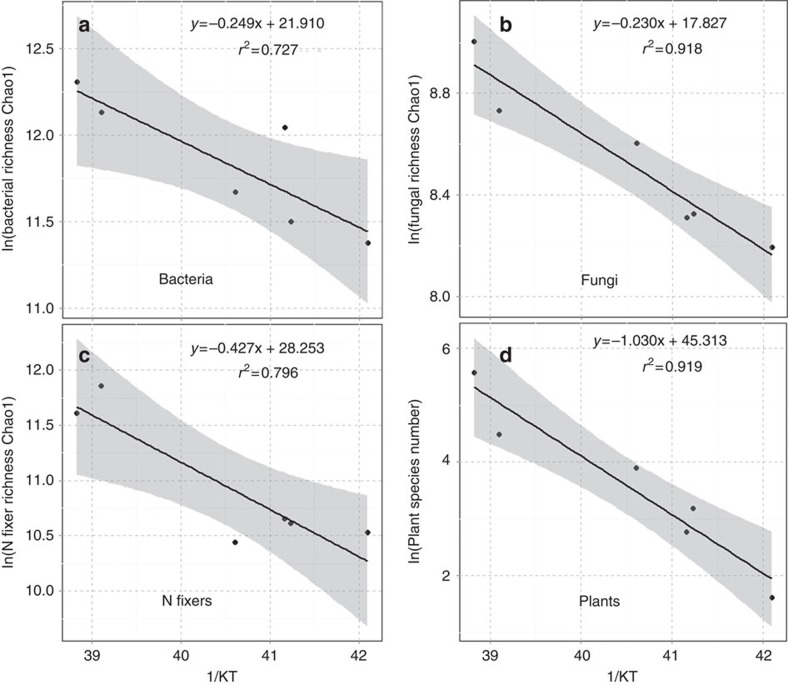
Relationships between taxon richness of individual locations and temperature. The sequences from all 21 samples in a site were pooled together and used for estimating theoretical Chao1 value for a given site at the taxonomic resolution of 97%. The natural log of the estimated Chao1 values were used for analysing the relationships between taxon richness and temperature, which was expressed as the inverse of annual mean temperature in degree of kelvin. Line represents least squares regression fit and the shaded area represents their 95% confidence limits. (**a**) Bacteria based on 16S rRNA gene; (**b**) Fungi based on ITS; and (**c**) N fixers based on *nifH* gene. (**d**) Plants.

To apply MTE more directly to our data on forest soil microbial communities, we used the Chao1 estimator^35^ to quantify the diversity of microbes and used Boltzmann–Arrhenius plots to assess the functional dependence of diversity on temperature. The Chao1 estimator incorporates the number of single gene copies in a sample to estimate the total diversity, including rare genotypes that were not sampled. Boltzmann–Arrhenius plots of the natural logarithm of diversity as a linear function of inverse absolute temperature (Fig. 3; Supplementary Fig. 3) highlight the exponential effects of temperature: the slope of such plots with sign reversed gives ‘activation energy' as a quantitative measure of temperature dependence.

Three different commonly used statistical models^29^, that is, a linear model^11^, a quadratic polynomial model^36^ and a piecewise relationship model^37^, were used to estimate the relationships between temperature and taxon richness. While there were no differences between linear and quadratic models for both 16S and ITS genes in terms of AIC, the linear model for *nifH* gene fit the data better than the quadratic and piecewise relationship model (Supplementary Table 9). Thus, the linear model was selected in this study. Our statistical analyses identified strong Boltzmann exponential relationships between the log-transformed Chao1-estimated taxon richness from individual samples and the reciprocal temperature (1/*kT*) for bacteria (Supplementary Fig. 3A), fungi (Supplementary Fig. 3B); and N fixers (Supplementary Fig. 3C). Temperature explained 42.0–65.1% (*r*^2^ values) of the variations in microbial diversity (Supplementary Fig. 3), which are consistent with those observed in plant communities^12^.

Next, to directly compare the estimated activation energies for microbes and plants, the sequences from all 21 samples in a site were pooled together and used for estimating the theoretical Chao1. For the plant diversity data, because of the differences of sampling strategies, richness data were only available for five 0.1-ha plots from each of the six sites. For microbes, in support of predictions from MTE the Boltzmann (linear negative) relationships between log-transformed Chao1-estimated taxon richness from individual sites and the reciprocal temperature (1/*kT*) for bacteria (Fig. 3a); fungi (Fig. 3b); and N fixers (Fig. 3c) explaining between 72.7–91.8% of the variation in taxon richness (Fig. 3). For plants, we also found a strong linear relationship (Fig. 3d; *r*^2^=0.919).

In this study, the estimated activation energies from the Boltzmann–Arrhenius plots varied considerably among the different genes. For instance, at the 97% taxa identity cutoff, the fitted slopes revealed that the *E*_a_ values varied between 0.184–0.249 for 16S rRNA genes, 0.169–0.230 for ITS and 0.427–0.467 for *nifH* gene (Table 2). The higher *E*_a_ values for *nifH* is most likely due to higher taxonomic resolutions of this functional gene marker compared with the ribosomal phylogenetic gene markers. In comparisons of the same gene, *E*_a_ values increased also with increased taxonomic/genetic resolution as expected (Table 2). For instance, the slopes for N-fixing bacteria increased from 0.411 to 0.467, when similarity cutoffs increased from 95 to 97% (Table 2). The *E*_a_ values determined were more or less consistent with that reported in zooplankton (*E*_a_=0.26)^38^, but quite lower than that in phytoplankton (*E*_a_=1.0)^29^.

Across all groups of microbes and at all levels of similarity resolution, the temperature dependence of microbial diversity was considerably less than that previously reported for plant and animal species diversity. The steepest temperature dependence for microbes at our study sites was *E*_a_=0.467 for N fixers using the *nifH* gene at 97% similarity (Table 2). This is half of that for tree species diversity at our same study sites (*E*_a_=1.030; Fig. 3d). The magnitude of variation with temperature and latitude was also considerably less than that reported for species diversity of most invertebrates and vertebrates^10^. Our results support the emerging generalization that the diversity of microbes and macrobes increases exponentially with temperature, but there is no ‘canonical' temperature dependence of species diversity^12^,^36^.

Our study suggests two alternatives, but not mutually exclusive, hypotheses for the observed lower-temperature dependence for diversity of forest soil microbes across the wide range of environmental temperatures. On one hand, the measured ‘activation energies' increase with the degree of taxonomic and phylogenetic resolution. It is certain, from the number of singleton OTUs in all of our samples and the current cutoffs at 95 and 97% identity, that we have sampled only a fraction of the genotypes present in the soils at our study site. Consistent with this hypothesis, we have shown that the apparent temperature dependence of diversity increases with increased phylogenetic resolution and sampling coverage. Fuhrman *et al*.^27^ analysed geographic variation in diversity of marine planktonic bacteria, and found a pattern with temperature and latitude generally comparable to those reported here for terrestrial soil bacteria. It remains to be seen whether, with much more complete sampling and higher resolution of genetic differences, the temperature dependence of microbes would approach and perhaps even exceed that of macroorganisms, that is, much larger plants and animals. On the other hand, the observed shallower temperature dependence of microbial diversity compared with tree species diversity that we have obtained across our sites may be due in part to the fact that microbe taxa are more widely distributed than plants and animals. Wider dispersal abilities could lead to lower spatial turnover rates among different sites and communities^23^. Nevertheless, the addition of more different forest sites sampled across different continents will help assess whether the results observed here are applicable to other ecosystems.

## Discussion

Since temperature is a primary driver of all biological processes, it is expected that temperature has important effects on ecological patterns and processes^7^. While the importance of temperature in controlling plant and animal communities is well documented, little is known in microbial communities, especially in soil environments. By examining 126 samples across a wide range of temperatures, our results showed that environmental temperature appeared to have a pervasive influence on variation in soil microbial diversity. This is the first demonstration that temperature plays a more primary role in shaping variation in microbial diversity in the forest soils than other proposed environmental drivers. This result is consistent with various previous analyses in soils, which showed temperature plays critical roles in controlling microbial growth and activities^34^. Importantly, our results show that the temperature gradient for microbes is not as steep as observed for macrobes. Microbial taxa richness increase across the observed global temperature range at a rate of 2–8 times lower than those observed for trees and many other animals, implying that temperature differentially influences species diversity in microbes and macrobes. Thus, while MTE provides a powerful framework for predicting broad large-scale biodiversity, further theoretical modelling development is needed to account for the unique characteristics of microorganisms, for example, extremely high diversity, large community size, high abundance, low extinction rates and long-distance dispersal.

Our findings have important implications for understanding and predicting ecological consequences of climate change. First, if temperature drives the increase in microbial diversity, we see from the tropics to an alpine forest, warming ecosystems should often become more diverse and active^27^, with enhanced processes—such as decomposition, nutrient cycling and carbon sequestration—that depend on this diversity^39^. Such changes in ecosystem function could, in turn, collectively shape feedbacks of ecosystems to climate warming. Also, the simple temperature-dependent Arrhenius relationships between temperature and microbial biodiversity could provide a quantitative framework for predicting how climate warming impacts diversity and ecosystem processes, though other factors (for example, pH, plant diversity and nutrient availability) should be incorporated into the MTE-based models^40^ to improve predictive accuracy. In a word, MTE-based kinetic models could provide powerful tools for projecting the effects of current and future climate warming on biodiversity and ecosystem processes^6^,^7^,^11^.

## Methods

### Site description and sampling

The following six forests long-term ecological research were selected for this study (Supplementary Table 1). Five belonged to the US National Science Foundation (NSF) long-term ecological research network: Niwot, Andrews, Harvard, Coweeta and Luquillo. A sixth, Barro Colorado Island, is administered by the Smithsonian institution. The selected sites provide variation in ecosystem type from boreal to tropical forest, in average annual temperature from 2.5 to 25.7 °C, and a rough gradient of latitude from 9–44° N (Supplementary Table 1). Moreover, Luquillo and Barro Colorado Island are two tropical forests, the former in the Carribean Sea, the other in the island of Panama. Hourly temperature and annual precipitation data were collected through the nearest weather stations on sites, and mean temperature and average annual precipitation were calculated thereafter.

A nested sampling design was implemented to examine the microbial diversity at each of six forest sites. At each site, we collected and homogenized nine surface soil cores (∼10-cm depth, Oakfield Apparatus Company model HA) from 21 individual square metre plots in the summer of 2002. The 21–1-m^2^ plots were laid out in a cross pattern (Fig. 1), with plots adjacent to 1, 10, 50, 100 and 200 m in each cardinal direction from a central 1-m^2^ plot. Soils were kept on ice in the field, then at −20 °C (LUQ, CWT, AND and NWT) or −80 °C (BCI and HFR) until shipped overnight on dry ice to the Institute for Environmental Genomics at the University of Oklahoma.

### Plant diversity

Plant species were surveyed by the Enquist lab using a modified ‘Gentry plot' methodology whereby five 0.1-ha Gentry plots were established within the 25-ha plot at each site. Each Gentry plot consisted of five 100 × 2 m transects separated by a distance of 8 m, so that each Gentry plot was located within a 42 × 100  m area. All plant stems >1-cm basal diameter that were rooted within the transects were censused and identified to species. For plants that extended outside of the transect boundaries, inclusion criteria varied by growth form: trees were censused if the centre of their stem base fell within the transect bounds, lianas were censused if rooted within the transect and hemiepiphytes were censused if any part of the aerial root of rhizome fell within the transect. Stems were tallied as separate individuals if they were not connected above ground or below ground within ∼10 cm of the soil surface.

### Soil chemistry

The soil moisture was measured by putting 1.5 g soil into 65 °C oven until constant weight was reached. The percentage of weight loss after oven dry to the original weight was calculated as soil moisture content (%). Soil pH was measured in soil suspension with a soil:water ratio of 1:2.5 (weight:volume) according to the standard protocol described previously^41^. The soil C and N contents were measured by a LECO TruSpec Carbon and Nitrogen Analyzer (LECO Corporation, St Joseph, MI) in the Soil, Water and Forage Analytical Laboratory at the Oklahoma State University (Stillwater, OK). In the same analytical laboratory, the soil NH_4_^+^, NO_3_^−^ contents extracted from soils with 1 M KCl based on the standard protocol described previously^42^ and measured by Lachat Quickchem 8500 series 2 instrument (Lachat, Loveland, CO).

### DNA extraction

Soil DNA was extracted using the grinding SDS-based DNA extraction method as previously described^43^. The quality was assessed based on spectrometry absorbance at wavelengths of 230, 260 and 280 nm (ratios of absorbance at 260/280 nm ∼1.8 and 260/230 nm >1.7) detected by a NanoDrop ND-1000 Spectrophotometer (NanoDrop Technologies). DNA concentration was measured by PicoGreen using a FLUOstar OPTIMA fluorescence plate reader (BMG LABTECH, Jena, Germany).

### Amplicon sequencing

For 16S rRNA genes, the V4 region was amplified with the primer pair 515F (5′- GTGCCAGCMGCCGCGGTAA -3′) and 806R (5′- GGACTACHVGGGTWTCTAAT -3′) combined with the Illumina adaptor sequence, a pad and a linker of two bases, and a barcode sequences on the reverse primers. PCR amplification was performed in 25 μl reactions containing 2.5 μl 10 × AccuPrime PCR buffer (including dNTPs) (Invitrogen, Grand Island, NY), 0.4 μM of both forward and reverse primers, 10 ng of template DNA and 0.2 μl AccuPrime High-Fidelity Taq Polymerase. Three replicates of amplifications were made for each sample and mixed after PCR amplification to minimize potential biases from amplification^44^. Thermal cycling conditions were as follows: initial denaturation at 94 °C for 1 min, followed by 30 cycles of 94 °C for 20 s, 53 °C for 25 s and 68 °C for 45 s, with final extension at 68 °C for 10 min

For *nifH* gene and fungal ITS sequencing, the phasing amplicon sequencing approach was used^45^. For *nifH*, an amplicon of 302 bp (excluding the primers) was targeted using the primers: Pol115F, TGCGAYCCSAARGCBGACTC and Pol457R, ATSGCCATCATYTCRCCGGA . For fungal ITS, an amplicon of 309 bp (not including the primers) in ITS2 region was targeted using the primers: gITS7F, GTGARTCATCGARTCTTTG and ITS4R, TCCTCCGCTTATTGATATGC 46.

A two-step PCR was performed for ITS and *nifH* amplicon sequencing to avoid extra PCR bias that could be introduced by the added components in the long primers used for PCR library preparation^45^. Phasing primers were designed and used in the second step of the two-step PCR. Spacers of different length (0–7 bases) were added between the sequencing primer and the target gene amplification to randomize base position during sequencing^45^. To ensure that the total length of the amplified sequences remain constant with the primer set used, the forward and reverse primers were used in a complementary manner so that all of the extended primer sets have exactly seven extra bases as the spacer for sequencing phase shift^45^. Both forward and reverse phasing primers include the Illumina adaptor, the Illumina sequencing primer, a spacer, and the target gene primer and a barcode of 12 bases in the reverse primer between the sequencing primer and the adaptor. In the two-step PCR, the first round PCR was carried out in a 50 μl reaction containing 5 μl 10 × PCR buffer II (including dNTPs), 0.5 U high-fidelity AccuPrimeTaq DNA polymerase (Life Technologies), 0.4 μM of both forward and reverse target only primers and 10 ng soil DNA. Reactions were performed in triplicate and the sample amplification program described above was used except that only 10 cycles were performed, and the annealing temperature was 56 °C for ITS and 62 °C for *nifH*. The triplicate products from the first round PCR were combined, purified with an Agencourt AMPure XP kit (Beckman Coulter, Beverly, MA, USA), eluted in 50 μl water and aliquoted into three new PCR tubes (15 μl each). The second round PCR used a 25 μl reaction containing 2.5 μl 10 × PCR buffer II (including dNTPs), 0.25 U high-fidelity AccuPrime Taq DNA polymerase (Life Technologies), 0.4 μM of both forward and reverse phasing primers and 15 μl aliquot of the first round purified PCR product. The amplifications were cycled 20 times following the above program. Positive PCR products were confirmed by agarose gel electrophoresis. PCR products from triplicate reactions were combined and quantified with PicoGreen.

PCR products from samples to be sequenced in the same MiSeq run (generally 3 × 96=288 samples) were pooled at equal molality. The pooled mixture was purified with a QIAquick Gel Extraction kit (Qiagen Sciences, Germantown, MD, USA) and re-quantified with PicoGreen. Sample libraries for sequencing were prepared according to the MiSeq Reagent Kit Preparation Guide (Illumina, San Diego, CA, USA) as described previously^45^,^47^. First, the combined sample library was diluted to 2 nM. Then, sample denaturation was performed by mixing 10 μl of the diluted library and 10 μl of 0.2 N fresh NaOH, and incubated 5 min at room temperature. A measure of 980 μl of chilled Illumina HT1 buffer was added to the denatured DNA and mixed to make a 20 pM library. Finally, the 20 pM library was further adjusted to the desired concentration (∼12 pM) for sequencing using chilled HT1 buffer. The library for sequencing was mixed with a certain proportion of a Phix library of the same concentration to achieve a 10% Phix spike.

A 300-cycle v1 (for 16S ribosomal DNA, rDNA) or 500-cycle v2 (for ITS or *nifH*) MiSeq reagent cartridge (Illumina) was thawed for 1 h in a water bath, inverted 10 times to mix the thawed reagents and stored at 4 °C for a short time until use. For 16S rDNA sequencing, customized sequencing primers for forward, reverse and index reads were added to the corresponding wells on the reagent cartridge before being loaded as described previously^47^. Sequencing was performed for 151, 12 and 151 cycles (for 16S rDNA), or 251, 12 and 251 cycles (for ITS and *nifH*) for forward, index and reverse reads, respectively.

### Sequence preprocessing

The raw reads of 16S, ITS and *nifH* genes were collected in Miseq sequencing machine in fastq format. Their forward and reverse directions, and barcodes were generated into separated files. First, the spiked PhiX reads were removed by using BLAST against PhiX genome sequence in *E* value <10^−5^. Second, the reads were assigned to samples according to the barcodes in the barcode file with up to one mismatch allowed. For both 16S and ITS, forward and reverse reads of same sequence with at least 10 bp overlap and <5% mismatches were combined, as single sequence by using FLASH program^48^, while the minimum overlap length for *nifH* was set to 50 bp. Any joined sequences with an ambiguous base, or <240 bp for 16S, <200 bp for ITS and <229 bp for *nifH* were discarded. Besides, the Btrim program^49^ with threshold of QC >20 over 5-bp window size was used to further filter the unqualified sequences. Thereafter, U-CHIME^50^ was used to remove chimeras by searching against green reference data set^51^ for 16S data set, against UNITE/QIIME released ITS reference (http://qiime.wordpress.com/2012/11/27/uniteqiime-12_11-its-reference-otus-now-available-alpha-release/) for ITS data set and against *nifH* database released by Zehr Laboratory (http://pmc.ucsc.edu/~wwwzehr/research/database/) for *nifH* data set.

OTUs were classified using UCLUST with different similarity levels^52^ for all 16S, ITS and *nifH* genes. Thereafter, the reads of OTUs were re-assigned back to their samples and a big matrix with 126 samples as columns and all OTUs as rows was generated for each data set. Since, reliable taxonomic assignments (OTU annotations) for both ITS and *nifH* were still unavailable, we only got taxonomic annotation for 16S data set through Ribosomal Database Project (RDP) classifier^53^ with minimal 50% confidence score. All of the sequences were also reanalyzed using the recent program UPARSE, which was developed by the same author^54^. The main differences of these two programs are the algorithms for read quality filtering and the chimera filtering. For the 16S data, about five times of less OTUs were obtained with UPARSE than UCLUST. Although the OTU numbers obtained by these two programs were markedly different, but relationships between the OTU richness, and climate and soil variables were not changed significantly (data not shown), no matter what programs were used. Since, UPARSE requires good reference database by using UPARSE-REF algorithm to remove chimeras, reliable OTU classification could not be obtained with *nifH* data because of lack of comprehensive reference database. Thus, to be consistent, all of the sequence analysis results reported were based on UCLUST approach in this study.

### Richness estimation and diversity calculation

In this study, we employed two ways to measure the richness through OTU table. First, the number of species for each sample was estimated by using Chao1 value^35^, that is,


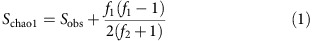


where *S*_obs_ is the number of OTU observed in this sample and *f*_1_ and *f*_2_ are the numbers of singleton and doubleton OTUs. For each sample, *f*_1_ and *f*_2_ were counted through each column of OTU matrix. For each site, the abundance of every OTU was summed by all 21 replicates and then *S*_obs_, *f*_1_ and *f*_2_ were measured through the pooled OTU abundances. Chao1 values represented the estimated species richness in samples or sites. Another easier way to compare the richness of different communities was to use observed richness through equal amount of sampling size. We randomly picked up the sequences from larger samples until they were reached the same size as the smallest sample. The minimum numbers of resampled sequences were 25,901 for 16S rRNA, 13,688 for ITS and 16,000 for *nifH* genes.

The taxonomic diversity in this study was measured by Shannon–Weaver index, that is,


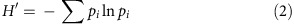


where *p*_*i*_ is the proportion of the *i*th OTU abundance to total abundance in certain sample. Unlike richness and Faith's phylogenetic diversity, the Shannon index took species abundance into account and thus it reflected both richness and abundance distributions.

Another taxonomic diversity index is inverse Simpson index, that is,


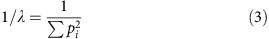


where *p*_*i*_ is still the proportion of the *i*th OTU abundance to total abundance in certain sample.

The phylogenetic diversity was measured by two approaches. The first is to use Faith's approach^55^, which is the sum of the total phylogenetic branch length of detected OTUs in each sample. To calculate this, phylogenetic trees were firstly generated for 16S, ITS and *nifH* data sets, respectively. For 16S genes, a representative sequence was selected from each OTU. The selected representative sequences were aligned using PyNAST^56^ against with GreenGene 16S Core Set alignment. For ITS sequences, the selected OTU representative sequences were self-aligned by MUSCLE alignment program^57^. For *nifH* sequences, the OTU representative sequences were aligned using Mothur software^58^, with default kmer searching option (http://www.mothur.org/wiki/Align.seqs). All of the trees were constructed using by FastTree2 program^59^. After all trees were constructed, the Faith's phylogenetic diversity was calculated using Picante package in R^60^.

The second approach is based on NRI. NRI was calculated based on abundance-weighted mean pairwise phylogenetic distance (MPD)^61^ and the manual of Phylocom^62^:


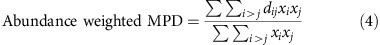



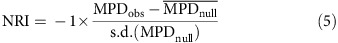


where *d*_*ij*_ is phylogenetic distance between observed taxa *i* and *j*, *x*_*i*_ and *x*_*j*_ are relative abundances of taxa *i* and *j*, MPD_obs_ is observed MPD, and 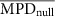 and s.d.(MPD_null_) are the mean and s.d. of MPD in the null communities. We used the null model called phylogeny shuffle to generate 1,000 null communities as described in Webb *et al*.^62^. The bigmemory^63^ and snow^64^ packages in R were used to harness the large data sets when calculating NRI.

### Statistical methods

Pearson correlations were used to reveal the linear dependence between two variables and the correlations between microbial diversity and sample traits. The significance of Pearson correlation is inferred through the Student's *t*-distribution with degrees of freedom *n*-2. Since, some environmental variables had only single value for each site (for example, mean temperature, annual precipitation and so on), to keep consistent, the mean values for all other variables with multiple measurements (for example, pH, soil moisture total carbon and total nitrogen) were obtained and used for subsequent statistical analysis.

The goodness of fit with linear and non-linear models was assessed by the coefficient of determination (*r*^2^) and AIC. Coefficient of determination is defined as,


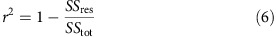


where *SS*_res_ is the sum of squares of residuals and *SS*_tot_ is the total sum of squares. Since the residuals can be considered as variance of the model's errors, the term *SS*_res_/*SS*_tot_ represents the unexplained proportion and thus *r*^*2*^ is the proportion of the explained variance of the linear or non-linear model. Meanwhile, the AIC was also used to determine which model fits best the experimental data: AIC=−2 × ln(*L*)+2*n*, where *L* is the probability of the data given a model and *n* is the number of parameters^65^. The smaller AIC value, the better model fits.

Mantel test was used for testing correlations between two distance matrixes, and partial Mantel were used to determine the contributions of various factors to explain community variations. The significance of the test was measured by random permutations^66^. For environmental variables, the best combination of environmental variables was selected by BioENV with ranked correlations^28^. The selected environmental variables were normalized to zero mean and one unit s.d. The distances among samples were calculated based on Euclidean dissimilarity. For community data sets, Bray–Curtis distance was used^67^. Partial Mantel tests were performed in R using vegan package^68^.

### Fittings of MTE

One of the major predictions of MTE is the number of species increases exponentially with environmental temperature^7^. We tested this hypothesis by using Chao1 estimated microbial species richness and annual average temperatures across six sites. The linear model^11^ is,


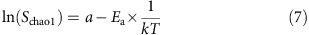


where *k* is Boltzmann's constant, *T* is absolute temperature in kelvin (K)^7^ and *a* is the intercept of this linear model. The *E*_a_ also called as the activation energy that equals the inverse number of slope in the linear regression.

Two other statistical models, a quadratic polynomial model^36^ and a piecewise relationship model^69^, were also used for estimating the relationships between temperature and taxon richness. Model selection was based on AIC, followed by explained variance (*r*^2^) and parameter significance (*P* values). If the differences of AIC values between two models are <2, these models are considered competitive^29^.

### Data availability

The OTU tables of 16S, ITS and *nifH* sequences that support the findings of this study are available in the institute website, http://ieg.ou.edu/4download. The raw sequencing data have been deposited in the NCBI Sequence Read Archive under accession code PRJNA308872.

## Additional information

**How to cite this article:** Zhou, J. *et al*. Temperature mediates continental-scale diversity of microbes in forest soils. *Nat. Commun.* 7:12083 doi: 10.1038/ncomms12083 (2016).

## Supplementary Material

Supplementary Information Supplementary Figures 1-3 and Supplementary Tables 1-9

## Figures and Tables

**Table 1:** Partial Mantel test to evaluate the relative importance of soil and site variables in determining microbial community structure.

**Factors**	**Control for**	**16S**		**ITS**		***nifH***	
		***r***_**M**_	***P*-value**	***r***_**M**_	***P*-value**	***r***_**M**_	***P*-value**
Temperature	Precipitation, pH, TN, TC	**0.674**	**0.001**	**0.284**	**0.001**	**0.177**	**0.001**
Precipitation	Temperature, pH, TN, TC	0.565	0.001	0.324	0.001	0.299	0.001
pH	Temperature, Precipitation, TN, TC	**0.179**	**0.001**	**0.117**	**0.001**	**0.150**	**0.001**
TN, TC	Temperature, Precipitation, pH	0.003	0.423	0.298	0.001	0.273	0.001

Since, the relative roles of pH and temperature on controlling microbial communities are controversial, their values were particularly bolded in the table. TN, total nitrogen; TC, total carbon.

**Table 2:** Summary of activation energy (*E*_a_).

T**axonomic groups**	**Taxonomic resolution (%)**	Individual samples*		**Pooled richness per site**†	
		***r***^**2**^	***E***_**a**_	***r***^**2**^	***E***_**a**_
Bacteria (16S)	97	0.420	0.184	0.727	0.249
	99	0.337	0.175	0.689	0.237
Fungi (ITS)	95	0.323	0.134	0.946	0.248
	97	0.419	0.169	0.918	0.230
N fixers (*nifH*)	95	0.613	0.411	0.716	0.288
	97	0.651	0.467	0.796	0.427

^*^Theoretical OTUs were estimated based on individual samples (126 samples) using Chao 1 estimator. Linear regressions were performed between natural log-transformed theoretical taxon richness and the reciprocal temperature (1/*kT*).

^†^Theoretical OTUs were estimated based on individual sites by pooling all sequence reads together from 21 samples, and then Chao 1 estimators for individual sites were derived from all of the pooled sequence reads.
