# Supplementary material for: Temperature mediates continental-scale diversity of microbes in forest soils
Source: Nat Commun. 2016 Jul 5;7:12083. doi: 10.1038/ncomms12083 (PMC4935970; doi:10.1038/ncomms12083)
Supplement: Supplementary Information — Supplementary Figures 1-3 and Supplementary Tables 1-9 [file ncomms12083-s1.pdf]

Supplementary Figures

(A) 16S rRNA genes (Bacteria)

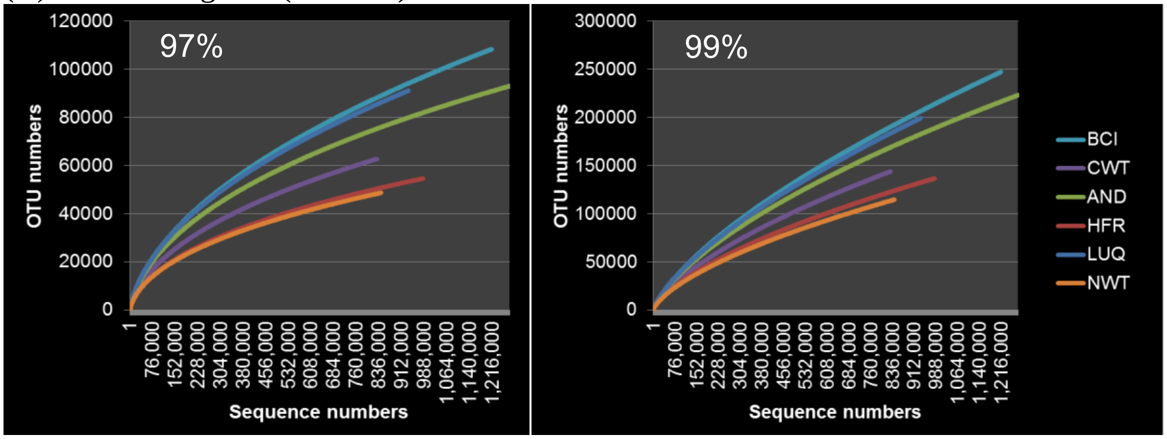

(B) ITS (Fungi)

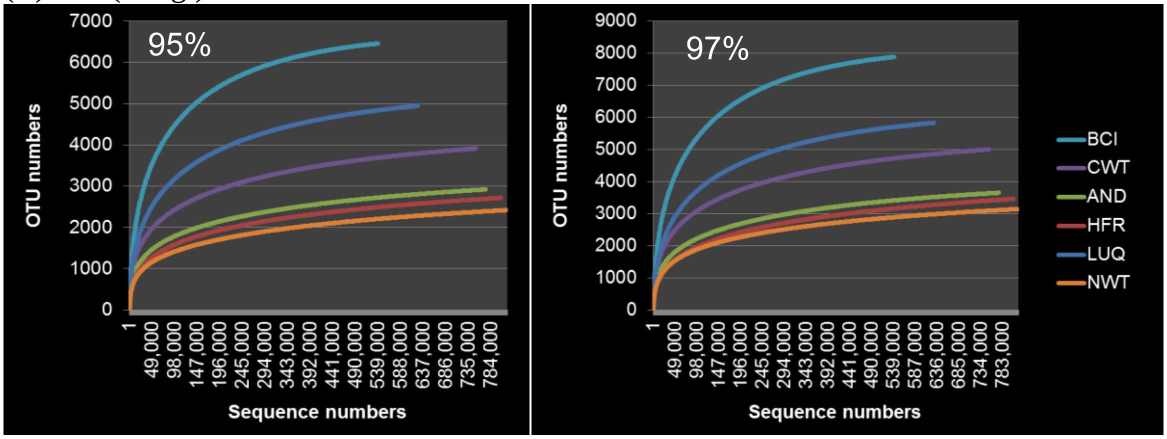

(C) nifH genes (N fixers)

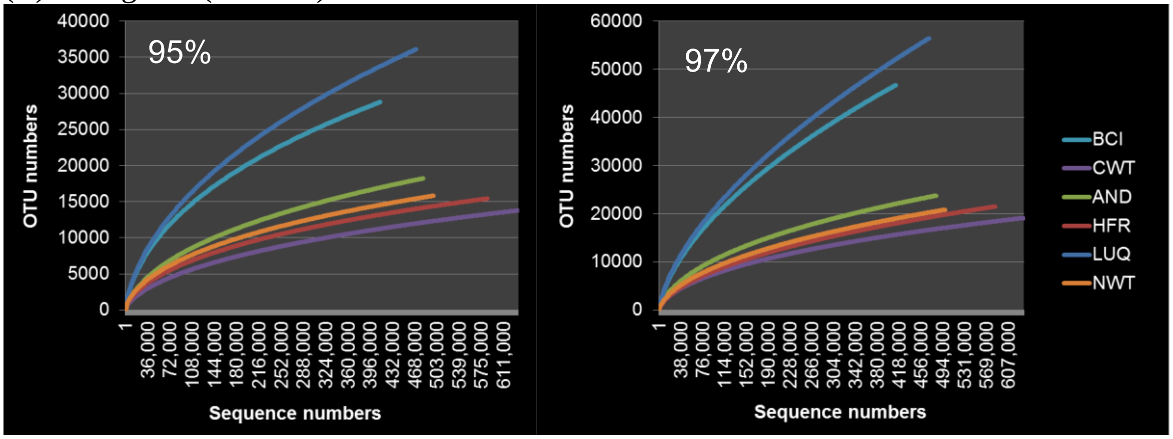

Supplementary Fig. 1. Rarefaction analysis of OTUs at different taxonomic resolutions.

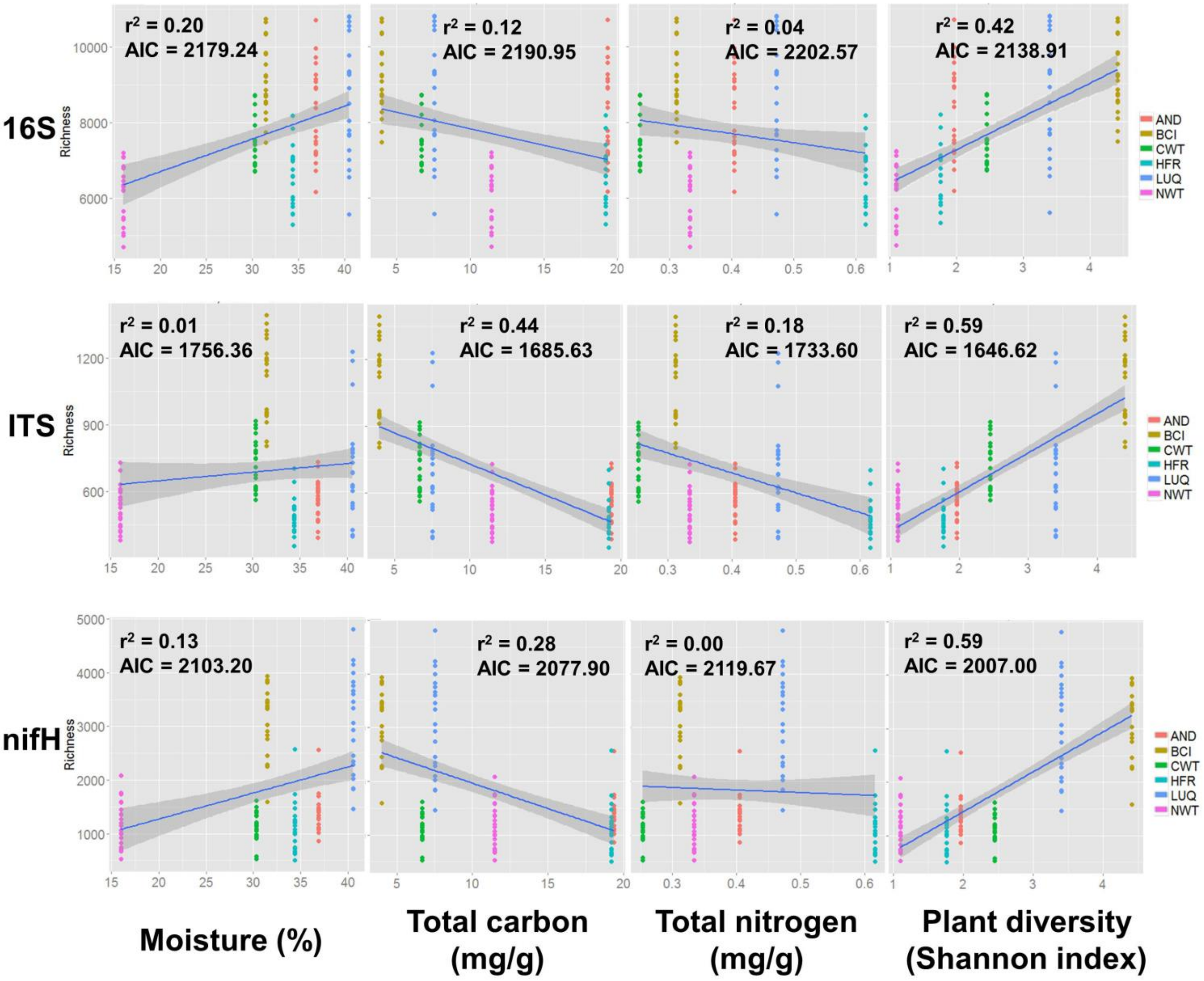

Supplementary Fig. 2. Relationships between taxon richness and several other soil and climate variables. Line represents least squares regression fit and shaded area represents 95% confidence. The relationships of taxon richness with temperature, pH and moisture were seen in Fig. 1. The relationships of microbial biodiversity (taxon richness, Shannon diversity and phylogenetic diversity) to all soil, climate and plants were summarized in Table S5.

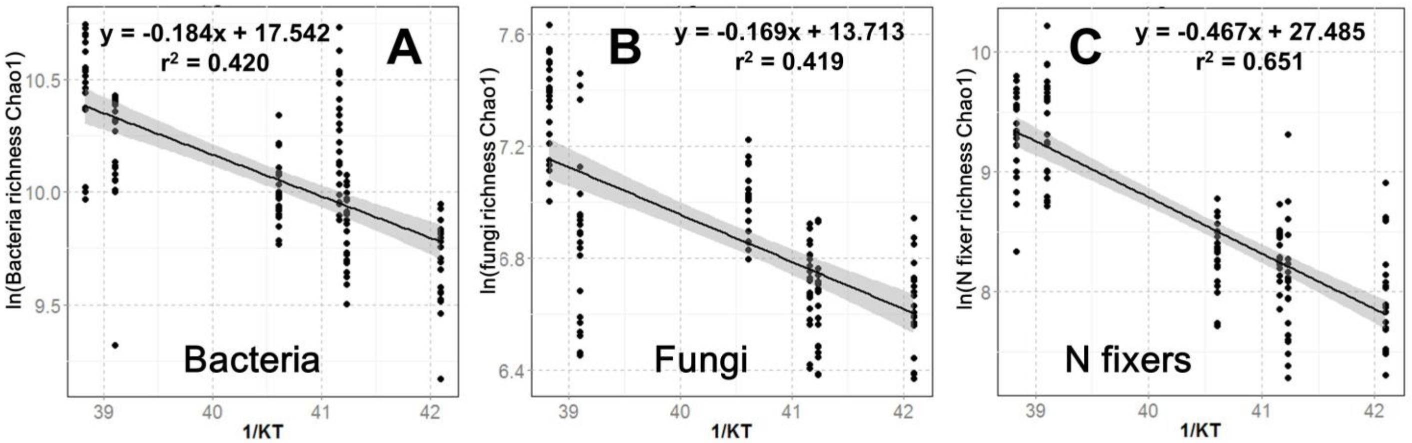

Supplementary Fig. 3. **Relationships between taxon richness of individual samples and temperature**. The sequences of individual samples in a site were used for estimating theoretical Chao1 value for a given sample at the taxonomic resolution of 97%. The natural log of the estimated Chao1 values of individual samples were calculated for examining the relationships between taxon richness and temperature, which was expressed as the inverse of annual mean temperature in degree of Kelvin. Line represents least squares regression fit and the shaded area represents their 95% confidence limits (A) Bacteria based on16S rRNA gene; (B) Fungi based on ITS; and (C) N fixers based on nifH gene. The relationships between microbial taxon richness of individual sites and temperature were seen in Fig. 3.

Supplementary Tables

Supplementary Table 1. Summary of soil and climate characteristics for the 6 sampling sites.

Sites[a]	Latitude	Elevation (m)	Mean temperature (°C)	Precipitation (mm)	Soil moisture[b] (%)	Soil pH	Total carbon content (mg/g)	Total nitrogen content (mg/g)	Plant species number[c]
BCI	9.16	157	25.71	2383.0	31.43±6.77	5.87±0.42	3.99±2.09	0.31±0.12	263
LUQ	18.32	386	23.62	3069.2	40.53±4.42	5.06±0.39	7.54±3.05	0.47±0.17	93
CWT	35.05	864	12.62	1853.8	30.28±6.05	4.72±0.37	6.68±4.07	0.25±0.08	49
AND	44.23	860	8.94	1587.4	36.88±13.79	5.28±0.64	19.36±13.87	0.40±0.18	17
HFR	42.54	356	8.27	1128.7	34.35±10.45	3.84±0.29	19.21±8.60	0.62±0.29	25
NWT	40.04	3,186	2.50	481.6	16.00±8.74	4.83±0.37	11.46±7.03	0.33±0.18	5

[a]The symbols for these sites are: BCI, Barro Colorado Island, Panama; LUQ: Luquillo, El Yunque National Forest, Puerto Rico; CWT: Coweeta Hydrological Laboratory, North Carolina; AND: H.J. Andrews Experimental Forest, Oregon; HFR: Harvard Forest, Massachusetts; NWT: Niwot Ridge, Colorado.
[b]Average individual soil variables (moisture, pH, total C and N) over 21 samples.
[c]Plant species is the total of five 0.1 ha Gentry plots within the 25 ha plot at each site.

Supplementary Table 2. Pairwise Pearson correlations (r values, n=6) between major soil and site variables. For the soil variables, the site averages among 21 samples were used for calculating correlations.

	Elevation	Mean temperature	Precipitation	Soil moisture	Soil pH	Total carbon	Total nitrogen	Plant richness
Latitude	0.447	-0.934**	-0.774	-0.252	-0.656	0.816*	0.252	-0.916**
Elevation		-0.706	-0.722	-0.876*	-0.106	0.088	-0.320	-0.511
Mean temperature			0.929**	0.571	0.587	-0.673	-0.106	0.852*
Precipitation				0.740	0.482	-0.551	-0.044	0.608
Soil moisture					0.057	0.127	0.452	0.197
Soil pH						-0.550	-0.612	0.690
Total carbon							0.648	-0.688
Total nitrogen								-0.282

* $p<0.05$
** $p<0.01$
*** $p<0.001$

Supplementary Table 3. **Summary of sequence statistics**

MiSeq sequencing	Targets	Total base pairs (bp)	bp per sample	Total No. of reads	Average No. of reads per sample
16S-rRNA gene	Soil bacteria + archaea*	2.05 G	16.3 M	8.10 M	64±18 K
ITS (internal transcribed spacer)	Soil fungi	1.39 G	11.0 M	4.94 M	39±11 K
nifH (nitrogenase subunit H)	Nitrogen fixing bacteria	1.17 G	9.3 M	3.75 M	30±9 K

*Very small percentages of sequences from Archaea were obtained.

Supplementary Table 4. **OTU classifications at different cutoffs**.

**Gene targets**	Similarity thresholds	No of OTUs without resampling[a]	No of OTUs with resampling[b]
16S	0.97	257,867	188,620
	0.99	789,214	524,047
ITS	0.95	13,762	12,942
	0.97	18,442	17,239
nifH	0.95	102,941	87,938
	0.97	160,082	133,192

[a]Estimated numbers of OTUs based on all sequences obtained, without resampling.
[b]Estimated numbers of OTUs after sampling the minimum numbers of sequences from each sample. The minimum numbers of sequences resampled are 25,901 for 16S rRNA genes, 13,688 for ITS and 16,000 for nifH.

Supplementary Table 5. Changes of microbial biodiversity with latitudes. The changes of taxon richness and Shannon diversity with latitudes were fitted with the linear model: y=*a* + *bx*. The significance of the relationships was evaluated based correlation (*r*) and probability (*p*) values.

**Taxonomic groups**[a]	**Taxon richness**		**Shannon diversity**	
	r	*p*	*r*	*p*
Bacteria (16S rRNA)	-0.550	$2.6x10^{-11}$	-0.389	$6.9x10^{-6}$
Fungi (ITS)	-0.744	$< 2.2x10^{-16}$	-0.454	$9.3x10^{-8}$
Nitrogen fixers (nifH)	-0.808	$< 2.2x10^{-16}$	-0.746	$< 2.2x10^{-16}$

[a]The taxonomic resolution used in this analysis is 97% for 16S rRNA gene and ITS, and 95% for nifH gene.

Supplementary Table 6. Summary of determination coefficients (R^2) from fitting a linear univariate model(y=a + bx) for predicting microbial biodiversity (taxon richness, Shannon diversity, phylogenetic diversity) as a function of various site and soil variables. Taxon richness, and Shannon diversity were calculated based on the observed sequences after resampling. Since the relative roles of pH and temperature on controlling microbial communities are controversial, their values were particularly bolded in the Tables.

(A) Taxon richness

Variables	16S (97%)		ITS (97%)		nifH (95%)	
	R^2	AIC	R^2	AIC	R^2	AIC
Latitude	0.30	2161.9	0.55	1656.4	0.65	1986.5
Elevation	0.22	2175.3	0.14	1738.8	0.17	2096.7
Annual average Temperature	**0.41**	**2141.8**	**0.48**	**1675.5**	**0.63**	**1994.8**
Annual precipitation	0.39	2144.8	0.27	1718.5	0.49	2034.6
Soil moisture	0.20	2179.2	0.01	1756.4	0.13	2103.2
pH	**0.35**	**2152.8**	**0.40**	**1693.7**	**0.34**	**2067.4**
Total Carbon	0.12	2190.9	0.44	1685.6	0.28	2077.9
Total Nitrogen	0.04	2202.6	0.18	1733.6	0.00	2119.7
Plant richness	0.30	2162.0	0.65	1626.0	0.51	2030.6

(B) Shannon diversity

Variables	16S (97%)		ITS (97%)		nifH (95%)	
	R^2	AIC	R^2	AIC	R^2	AIC
Latitude	0.15	89.4	0.21	270.3	0.56	303.6
Elevation	0.21	80.7	0.01	297.7	0.13	388.3
Annual average temperature	**0.28**	**68.5**	**0.16**	**278.1**	**0.53**	**312.3**
Annual precipitation	0.37	50.9	0.09	287.4	0.39	344.5
Soil moisture	0.29	66.5	0.00	299.3	0.11	391.5
pH	**0.19**	**84.1**	**0.20**	**271.1**	**0.35**	**350.9**
Total carbon	0.04	105.0	0.23	265.7	0.18	381.8
Total nitrogen	0.00	109.6	0.16	277.8	0.00	406.2
Plant richness	0.11	95.7	0.22	268.7	0.46	328.8

(C) Inverse Simpson diversity

Variables	16S (97%)		ITS (97%)		nifH (95%)	
	R^2	AIC	R^2	AIC	R^2	AIC
Latitude	0.00	1817.0	0.19	1129.5	0.56	1400.3
Elevation	0.07	1807.7	0.01	1155.0	0.14	1485.0
Annual average temperature	**0.05**	**1811.2**	**0.14**	**1137.0**	**0.52**	**1411.6**
Annual precipitation	0.18	1792.6	0.08	1145.5	0.37	1445.6
Soil moisture	0.24	1782.6	0.00	1156.5	0.08	1492.7
pH	**0.00**	**1817.1**	**0.15**	**1136.5**	**0.28**	**1461.8**
Total carbon	0.00	1817.3	0.20	1129.1	0.23	1470.2
Total nitrogen	0.04	1812.0	0.09	1144.3	0.00	1503.7
Plant richness	0.02	1815.0	0.19	1130.6	0.46	1426.1

(D) Faith's phylogenetic diversity

Variables	16S (97%)		ITS (97%)		nifH (95%)*	
	R^2	AIC	R^2	AIC	R^2	AIC
Latitude	0.36	1383.7	0.69	1280.7	0.04	1444.3
Elevation	0.23	1408.2	0.23	1394.7	0.00	1449.7
Annual average temperature	**0.47**	**1361.9**	**0.65**	**1296.2**	**0.04**	**1444.3**
Annual precipitation	0.44	1367.9	0.42	1360.1	0.04	1444.6
Soil moisture	0.20	1413.0	0.06	1420.4	0.02	1447.9
pH	**0.43**	**1370.8**	**0.43**	**1356.4**	**0.14**	**1431.4**
Total carbon	0.16	1418.9	0.48	1345.7	0.00	1449.6
Total nitrogen	0.05	1433.9	0.13	1410.4	0.00	1449.7
Plant richness	0.36	1384.9	0.74	1255.6	0.04	1445.4

*****The phylogenetic diversity of nifH gene fits poorly with all variables. Most likely, this is due to the difficulty in obtaining reliable sequence alignments for constructing phylogenetic tree to calculate phylogenetic diversity.

(E) NRI phylogenetic diversity

Variables	16S		ITS		nifH	
	R^2	AIC	R^2	AIC	R^2	AIC
Latitude	0.48	569.7	0.26	457.2	0.64	473.1
Elevation	0.19	625.8	0.14	477.2	0.18	574.7
Annual average temperature	**0.53**	**556.7**	**0.28**	**453.7**	**0.60**	**485.9**
Annual precipitation	0.47	572.4	0.24	460.6	0.44	526.9
Soil moisture	0.18	627.0	0.09	484.2	0.08	589.9
pH	**0.45**	**577.1**	**0.01**	**494.7**	**0.16**	**578.1**
Total carbon	0.17	627.6	0.14	477.2	0.40	535.5
Total nitrogen	0.02	649.7	0.02	493.7	0.01	599.4
Plant richness	0.41	585.9	0.16	474.6	0.48	518.2

Supplementary Table 7. Summary of determination (R^2) coefficients from fitting a non-linear univariate model($y=a+bx+cx^2$) for predicting microbial biodiversity (taxon richness, diversity) as a function of pH and temperature

(A) Taxon richness

Variables	16S (97%)		ITS (97%)		nifH (95%)	
	R^2	AIC	R^2	AIC	R^2	AIC
Annual average temperature	0.41	2142.7	0.51	1671.4	0.71	1966.2
Soil pH	0.36	2151.5	0.51	1669.3	0.40	2058.1

(B) Shannon index

Variables	16S (97%)		ITS (97%)		nifH (95%)	
	R^2	AIC	R^2	AIC	R^2	AIC
Annual average temperature	0.32	62.8	0.18	277.1	0.65	277.3
Soil pH	0.19	86.0	0.21	272.3	0.43	337.5

(C) Inverse Simpson index

Variables	16S (97%)		ITS (97%)		nifH (95%)	
	R^2	AIC	R^2	AIC	R^2	AIC
Annual average temperature	0.15	1798.41	0.17	1134.89	0.61	1388.30
Soil pH	0.08	1808.52	0.15	1137.48	0.36	1448.88

(D) ln(Faith's phylogenetic diversity)

Variables	16S (97%)		ITS (97%)		nifH (95%)*	
	R^2	AIC	R^2	AIC	R^2	AIC
Annual average Temperature	0.47	1363.4	0.67	1291.9	0.08	1441.8
Soil pH	0.44	1369.2	0.56	1326.0	0.14	1433.4

*****The phylogenetic diversity of nifH gene fits poorly with all variables. Most likely, this is due to the difficulty in obtaining reliable sequence alignments for constructing phylogenetic tree to calculate phylogenetic diversity.

(E) ln(NRI phylogenetic diversity)

Variables	16S (97%)		ITS (97%)		nifH (95%)*	
	R^2	AIC	R^2	AIC	R^2	AIC
Annual average Temperature	0.55	552.7	0.29	455.4	0.63	477.0
Soil pH	0.47	572.8	0.05	491.7	0.23	569.0

Supplementary Table 8. Summary of multiple linear regressions by including additional environmental or plant variables beyond temperature based on observed OTU richness.

Model	16S		ITS		nifH	
	AIC	R^2	AIC	R^2	AIC	R^2
richness ~ temperature	2141.84	0.41	1675.49	0.48	1994.82	0.63
richness ~ temperature+ precipitation	2142.06	0.41	1646.41	0.59	1993.64	0.64
richness ~ temperature+ pH	2127.36	0.48	1657.02	0.56	1989.34	0.65
richness ~ temperature+ pH + precipitation	2123.65	0.50	1633.54	0.64	1989.72	0.66
richness ~ temperature+ pH + precipitation + plant richness	2121.70	0.52	1621.91	0.68	1961.63	0.73
richness ~ temperature+ pH + precipitation + plant richness + total carbon + total nitrogen	2118.08	0.54	1614.83	0.70	1960.97	0.73

Supplementary Table 9. Summary of the fitting results to different statistical models to evaluate the relationships between temperature and taxa richness estimated with Chao1.

Model	16S		ITS		nifH	
	AIC	R^2	AIC	R^2	AIC	R^2
Linear	-191.36	0.42	-212.99	0.42	-88.47	0.61
Quadratic polynomial	-190.17	0.42	-211.44	0.42	-100.20	0.65
Piecewise relationship	-*	-	-	-	-102.44	0.66

* There is no turnover point observed
